# Soy-tomato enriched diet reduces inflammation and disease severity in a pre-clinical model of chronic pancreatitis

**DOI:** 10.1038/s41598-020-78762-9

**Published:** 2020-12-11

**Authors:** Debasmita Mukherjee, Mallory J. DiVincenzo, Molly Torok, Fouad Choueiry, Rahul J. Kumar, Anna Deems, Jenna L. Miller, Alice Hinton, Connor Geraghty, Jose Angel Maranon, Samuel K. Kulp, Christopher Coss, William E. Carson, Darwin L. Conwell, Phil A. Hart, Jessica L. Cooperstone, Thomas A. Mace

**Affiliations:** 1 James Comprehensive Cancer Center, The Ohio State University, Columbus, USA; 2 Department of Veterinary Biosciences, The Ohio State University, Columbus, USA; 3 Department of Food Science and Technology, The Ohio State University, Columbus, USA; 4 Division of Biostatistics, College of Public Health, The Ohio State University, Columbus, USA; 5 Tradichem SL (Innovation Center), Madrid, Spain; 6 College of Pharmacy, The Ohio State University, Columbus, USA; 7 Department of Surgery, The Ohio State University, Columbus, USA; 8 Division of Gastroenterology, Hepatology, and Nutrition, The Ohio State University Wexner Medical Center, 420 W 12th Ave., Columbus, OH 43210 USA; 9 Departments of Horticulture and Crop Science, The Ohio State University, Columbus, OH 43210 USA

**Keywords:** Inflammation, Pancreatitis

## Abstract

Chronic pancreatitis (CP) is a fibro-inflammatory syndrome in individuals who develop persistent pathological responses to parenchymal injury or stress. Novel therapeutic or dietary interventions that could lessen inflammation in this disease could significantly improve quality of life in patients with CP. Complex dietary foods like soy and tomatoes are composed of active metabolites with anti-inflammatory effects. Data from our group reports that bioactive agents in soy and tomatoes can reduce pro-inflammatory cytokines and suppressive immune populations. Additionally, our team has developed a novel soy-tomato juice currently being studied in healthy individuals with no toxicities, and good compliance and bioavailability. Thus, we hypothesize that administration of a soy-tomato enriched diet can reduce inflammation and severity of CP. C57BL/6 mice were injected intraperitoneally with 50 μg/kg caeurlein (7 hourly injections, twice weekly) for 6 weeks to induce CP. After 4 weeks of caerulein injections, mice were administered a control or a soy-tomato enriched diet for 2 weeks. Disease severity was measured via immunohistochemical analysis of pancreata measuring loss of acini, fibrosis, inflammation, and necrosis. Serum lipase and amylase levels were analyzed at the end of the study. Inflammatory factors in the serum and pancreas, and immune populations in the spleen of mice were analyzed by cytokine multiplex detection, qRT-PCR, and flow cytometry respectively. Infra-red (IR) sensing of mice was used to monitor spontaneous activity and distress of mice. Mice fed a soy-tomato enriched diet had a significantly reduced level of inflammation and severity of CP (*p* = 0.032) compared to mice administered a control diet with restored serum lipase and amylase levels (*p* < 0.05). Mice with CP fed a soy-tomato diet had a reduction in inflammatory factors (TNF-α, IL-1β, IL-5) and suppressive immune populations (myeloid-derived suppressor cells; MDSC) compared to control diet fed mice (*p* < 0.05). Infra-red sensing to monitor spontaneous activity of mice showed that soy-tomato enriched diet improved total activity and overall health of mice with CP (*p* = 0.055) and CP mice on a control diet were determined to spend more time at rest (*p* = 0.053). These pre-clinical results indicate that a soy-tomato enriched diet may be a novel treatment approach to reduce inflammation and pain in patients with CP.

## Introduction

Chronic pancreatitis (CP) is a fibro-inflammatory syndrome of the pancreas in individuals with genetic, environmental, and/or other risk factors who develop persistent pathological responses to parenchymal injury or stress^1^. Depending on etiology, CP patients also have an approximately 3–5 fold increased risk of developing pancreatic cancer^2–7^. CP patients experience symptoms of recurrent abdominal pain, nausea, and maldigestion, and disease complications that can include exocrine insufficiency, fat-soluble vitamin deficiency, metabolic bone disease, and diabetes mellitus^8–14^. To date, no clinical therapy is available to reverse the inflammatory damage associated with CP, so management consists of palliating symptoms and screening for and treating disease-related complications^15^. Novel therapeutic or dietary interventions that could lessen inflammation in this disease could significantly improve quality of life in patients with CP.

Complex dietary foods like soy and tomatoes are composed of active metabolites that can produce anti-inflammatory effects. The gut microbiota is responsible for breaking down soy into isoflavones (genistein, daidzein) and their metabolites, which our group and others have shown can have inhibitory effects on immunity and inflammation^16,17^. Data from our group reports that a soy-enriched diet can reduce pro-inflammatory cytokines and suppressive immune populations in prostate cancer patients, and soy isoflavones can reduce immune cell activation in vitro^17^. Tomatoes have been suggested to decrease systemic inflammation^18^ and reduce risk for a variety of inflammation-related chronic diseases, including cardiovascular disease^19^ and cancers^20^, in particular prostate cancer^21^. Carotenoids (lycopene) and flavonoids (naringenin, quercetin) are suggested as the bioactive agents responsible for the health benefits of tomatoes and have been shown to directly modulate inflammation^22–26^. Our team has developed a novel soy-tomato juice to use in patient trials which has previously been evaluated in healthy individuals for compliance, bioavailability, and effect on blood lipids^27^. Sensory characteristics were also evaluated and optimized, and the juice was found to be acceptable by consumers, which is critically important in developing functional foods. Use of bioactive foods, such as tomatoes and soy, as dietary interventions could lead to reduced inflammation and pain in patients with chronic disease. Thus, we hypothesize that administration of a soy-tomato enriched diet can reduce inflammation and severity of CP.

Here, we found that a soy-tomato enriched diet reduced overall inflammation, stromal fibrosis, and acinar destruction of pancreatic tissue. Additionally, the anticipated reduction in serum lipase and amylase levels was not observed in CP mice on the soy-tomato enriched diet, suggesting that acinar function had been maintained throughout the experiment. The overall inflammatory profile of the pancreas was reduced on the diet, where major genes that are associated with inflammation such as IL-6 and IL-1β showed an overall decreasing trend. In addition, CD68 and Ly6G, pro-inflammatory cell markers, were also decreased. Further, overall health of the mice were assessed by monitoring spontaneous activity. CP mice on the soy-tomato diet were more active and spent less time at rest compared to the control diet, suggesting improvement in overall health.

## Results

### Soy-tomato enriched diet reduces severity of chronic pancreatitis in a pre-clinical mouse model

**Figure 1:**
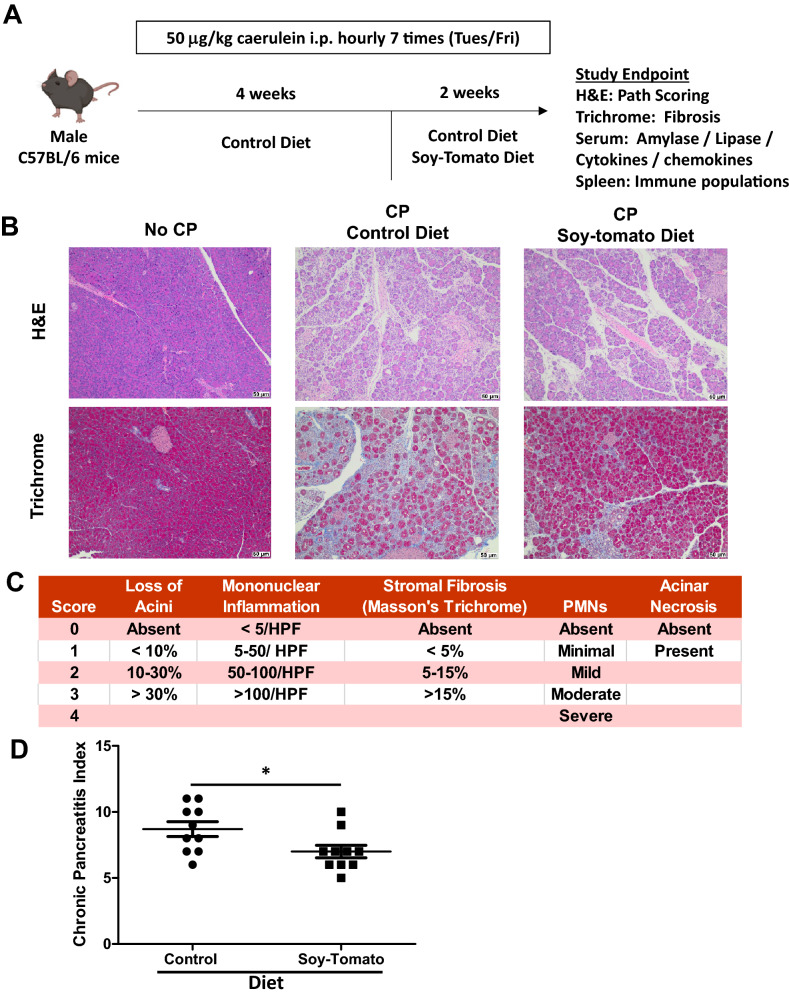
Soy-Tomato enriched diet reduces severity of chronic pancreatitis (CP). (**A**) C57BL/6 mice were injected intraperitoneally with 50 μg/kg caerulein (7 hourly injections, twice weekly) for 6 weeks. Mice across all groups were a fed a control diet from the start. After 4 weeks of injections, one group was fed a soy-tomato enriched dietary intervention (**B**) After 2 weeks of dietary intervention, severity of chronic pancreatitis was assessed from H&E and Trichrome stained pancreatic tissue at study endpoint. (**C**) Chronic pancreatitis index (score range 0–15) was expressed as a sum of scores for loss of acini, inflammation, necrosis, stromal fibrosis, polymorphonuclear cells (PMNs), and acinar necrosis; inflammation was measured for 10 random high power fields (HPF; 40× objective). Stromal fibrosis was evaluated in the entire pancreatic section on Masson's Trichrome-stained slides. (**D**) Chronic pancreatitis index scoring was quantified across all mice from each study group with healthy no CP mice having an index value of zero (not shown). N = 10 mice/group (*p < 0.05).

We were interested in testing whether a novel soy-tomato diet currently in clinical trials could modulate inflammation and disease severity of CP^27–29^. We utilized a well characterized murine model of caerulein-induced CP (Fig. 1A)^30^. Repetitive serial injections of caerulein primarily affect exocrine function of the pancreas leading to chronic pancreatitis with little overall effect on endocrine function^31^. We confirmed in a small cohort of mice that 4 weeks of injections of caerulein resulted in chronic pancreatitis (Supplemental Fig. 1). After mice developed CP (4 weeks), we fed a cohort of CP mice a soy-tomato diet designed to be identical to what we are currently using in our clinical trial (Supplementary Table 1) and a control cohort of CP mice was fed a control diet. A baseline group of mice received injections of PBS instead of caerulein and were fed a control diet. Mouse weights and food consumption were monitored throughout the study with no difference observed between the control and soy-tomato fed mice (Supplementary Fig. 2A and B). Following two weeks of administration of the soy-tomato diet, mice pancreata were collected and analyzed via H&E and Masson’s Trichrome staining to assess the severity of CP (Fig. 1B). As expected, the pancreata of baseline mice injected with PBS showed no pancreatitis and had healthy acinar cells without evidence of fibrosis. Mice with CP on the control diet had severe damage to their acinar cells and fibrosis of the pancreatic tissue. Mice with CP on the soy-tomato enriched diet had significantly less acinar cell loss and reduced interstitial and replacement fibrosis. Using a scoring system modified from a previously published model (Fig. 1C), we quantitatively determined that mice with CP fed a soy-tomato diet had a reduction in disease severity compared to mice fed a control diet (Fig. 1D; *p* = 0.032).

**Figure 2:**
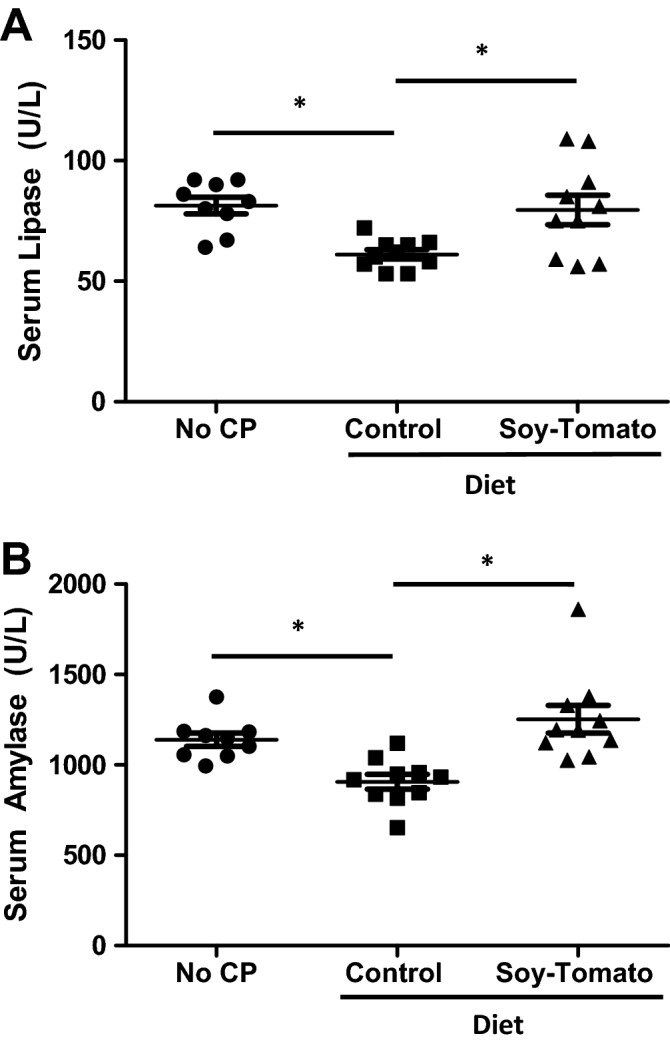
Serum lipase and amylase levels were restored in mice fed a soy-tomato enriched diet. C57BL/6 mice were injected intraperitoneally with 50 µg/kg caerulein (7 hourly injections, twice weekly) for 6 weeks. Mice across all groups were fed a control diet from the start. After 4 weeks of injections, one group was fed a soy-tomato enriched dietary intervention. At study endpoint, whole blood from mice was collected and serum was stored following centrifugation. Serum (**A**) lipase and (**B**) amylase were analyzed by the Alfa Wassermann VetAce analyzer. N = 5 mice/group (*p < 0.05).

### Serum lipase and amylase levels were restored in CP mice fed a soy-tomato enriched diet

Serum was collected at study endpoint and analyzed for serum lipase (Fig. 2A) and amylase (Fig. 2B) levels. The baseline mice with no pancreatitis had normal levels of lipase and amylase in their serum. Mice with CP on the control diet had significant reduction of their serum lipase (*p* = 0.007) and amylase (*p* = 0.018) enzyme levels which is attributed to the destruction of their acinar cells normally observed in a caerulein-induced pancreatitis model. Mice with CP fed a soy-tomato enriched diet had higher levels of lipase (*p* = 0.012) and amylase (*p* < 0.001) in their serum similar to the control mice without CP suggesting preservation of acinar function.

### Administration of a soy-tomato enriched diet significantly improves overall activity score and health of mice with chronic pancreatitis

**Figure 3:**
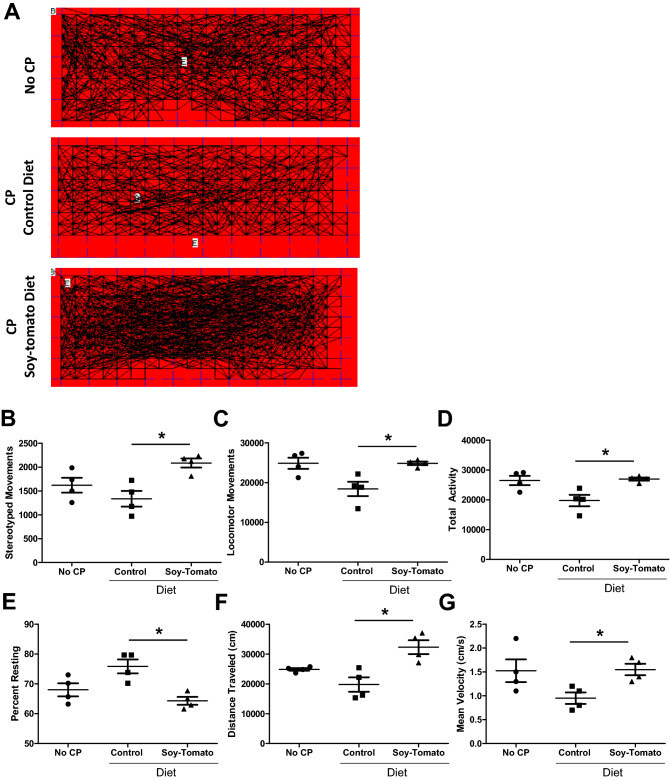
Soy-tomato fed mice with CP have increased physical activity and overall health of mice compared to control diet. Four days prior to study endpoint, activity levels of mice in each group (No CP, CP on control diet, and CP on a soy-tomato diet) were measured using an infrared-based sensing system. (**A**) Representative movement tracks for individual mice from each group for the first 2 h of the measurement interval. Quantified data for (**B**) stereotyped movement, (**C**) locomotor movement, (**C**) total activity, (**D**) percent of time resting, (**E**) distance traveled, and (**F**) mean velocity over a 6 h period was assessed. N = 4 mice/group, *p < 0.05.

To quantitatively assess if the improvements in histological and serum biochemical features of CP observed in soy-tomato diet-fed CP mice (Figs. 1, 2) were reflected in changes in general well-being of these mice, spontaneous physical activity was measured as an indicator of overall health using an infrared photobeam-based monitoring system (IR Actimeter, Panlab, Harvard Apparatus). Four days prior to study endpoint, mice were singly housed and activity parameters for each mouse were measured over 6 h (Fig. 3A). Compared to baseline mice without CP, mice with CP on the control diet showed reductions in stereotyped movement (Fig. 3B), locomotor movement (Fig. 3C), and total activity (Fig. 3D), along with an increase in the percentage of time at rest or inactivity (Fig. 3E). The consumption of the soy-tomato enriched diet reversed these CP-induced changes in activity parameters resulting in significantly elevated activity levels and reduced time at rest (*p* = 0.053). Additionally, total distance traveled (Fig. 3F) and mean velocity (Fig. 3G) were also improved for mice on a soy-tomato enriched diet. Overall, mice on a soy-tomato enriched diet had improved movement scores (*p* < 0.05), and were similar to mice without CP (Fig. 3B–G; *p* = 0.055). These data suggest a soy-tomato diet improved the overall physical performance and activity of mice with CP compared to mice with CP on a control diet, potentially due to an improvement in the overall health of these animals.

### Systemic and pancreatic tissue inflammatory profile was improved in the mice fed a soy-tomato enriched diet group

**Figure 4:**
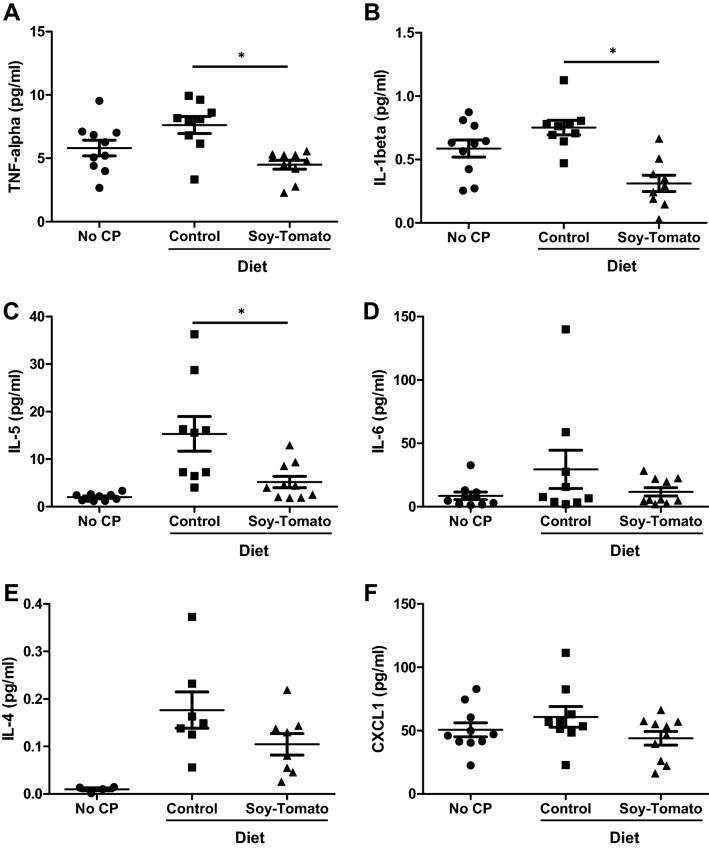
Reductions in systemic inflammatory cytokines in mice fed a soy-tomato diet. At the end of study, sera were collected from mice and analyzed by Meso Scale Discovery assay to determine systemic cytokine and chemokine expression. Serum levels of (**A**) TNF-alpha, (**B**) IL-1beta, (**C**) IL-5, (**D**) IL-6, (**E**) IL-4, and (**F**) CXCL1. N = 10 mice/group, *p < 0.05.

Next, we analyzed how a soy-tomato diet affects inflammatory cytokines and chemokines in mice with CP systemically in the blood and locally in the pancreatic tissue. We observed elevated serum TNF-alpha, IL-1β, IL-5, and IL-4 in mice with CP on a control diet (Fig. 4A–F). Feeding of the soy-tomato enriched diet to mice with CP significantly reduced the serum levels of some of these inflammatory cytokines, specifically TNF-alpha, IL-1β, and IL-5, compared to the control diet-fed CP animals (*p* < 0.05).

**Figure 5:**
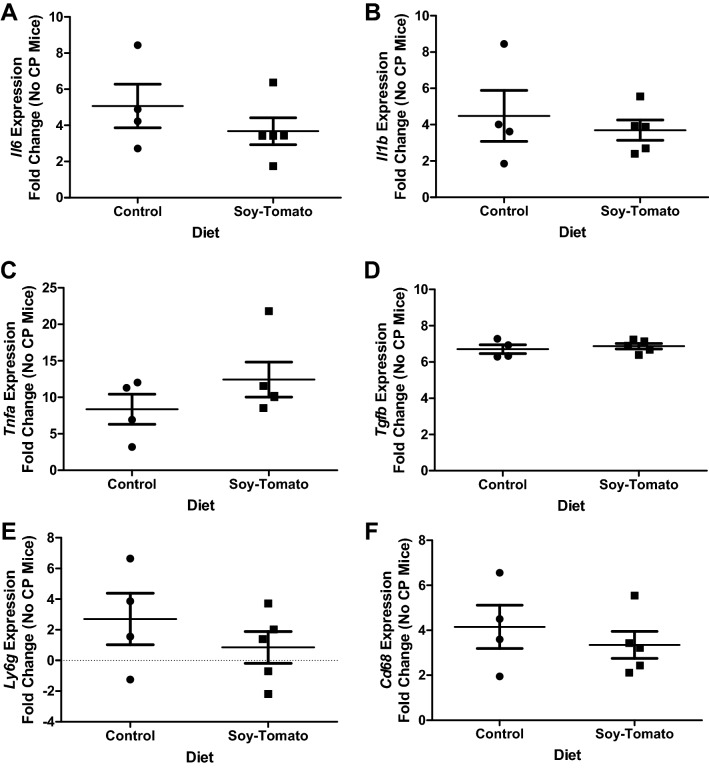
Soy-tomato diet reduces inflammatory gene expression in pancreatic tissue of mice with CP. At study endpoint, mice pancreata were harvested and placed in RNALater. Tissue samples were used to extract RNA and synthesize cDNA for RT-qPCR analysis of genes of interest. Gene expression was quantified and normalized to the 18S housekeeping gene and then normalized to healthy mice without CP. Expression levels of pro-inflammatory cytokines (**A**) IL-6, (**B**) IL-1beta, (**C**) TGF-alpha, (**D**) TNF-beta, and inflammatory cells such as (**E**) Ly6G and (**F**) CD68 were analyzed by RT-qPCR. N = 5 mice/group.

To identify factors locally within the pancreatic tissue, we performed qRT-PCR on RNA isolated from the pancreata of these mice. These data showed lower levels of expression of *Il6* and *Il1b* in the pancreatic tissue of mice with CP fed the soy-enriched diet compared to mice with CP fed a control diet (*p* = 0.413). Soy-tomato fed mice showed higher expression levels of *Tnfa* (*p* = 0.556) in their pancreatic tissue with no difference observed in *Tgfb* (Fig. 5D, *p*=0.730) levels. Interestingly, we also observed lower expression levels of *Ly6g* (*p* = 0.413) and *Cd68* (*p* = 0.556) genes indicating a potential reduction of granulocytic and macrophage populations within the tissue of soy-tomato fed mice. The results obtained from qRT-PCR for identification of factors locally were not statistically significant. However a larger experimental cohort size or longer duration of dietary intervention would perhaps lead to statistically significant changes locally as were observed systemically in the blood. Further, concentration of bioactive metabolites of soy and tomatoes in the pancreas are currently unknown in mice with CP. Future studies will investigate metabolomics and metabolite concentrations in the pancreas compared to serum levels as this may be explain cytokine differences in pancreatic tissue and blood.

**Figure 6:**
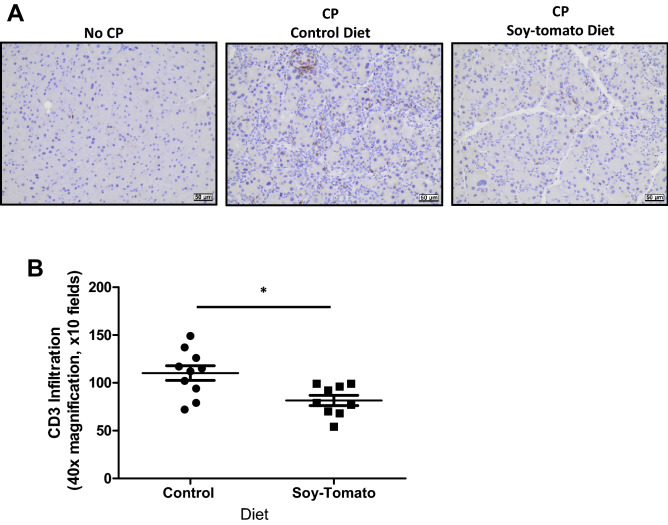
Reduction of infiltrating CD3 + T cells in mice with CP on a soy-tomato diet. Paraffin-embedded pancreatic tissue from mice in each of the cohorts were stained by immunohistochemistry for CD3 + T cells (Brown). (**A**) Tissue sections were imaged at 40× magnification and (**B**) immunopositive cells were quantified from 10 random fields (n = 10).

To further investigate the local inflammation within the pancreatic tissue, we stained the pancreata harvested at study endpoint for CD3^+^ T cells by immunohistochemistry (Fig. 6A). These results showed that the pancreata of CP mice fed a soy-tomato enriched diet contained significantly fewer numbers of CD3 + T cells than those of CP mice fed a control diet (*p* = 0.007).

### Soy-tomato dietary intervention reduces systemic inflammatory and suppressive immune populations in mice with CP

**Figure 7:**
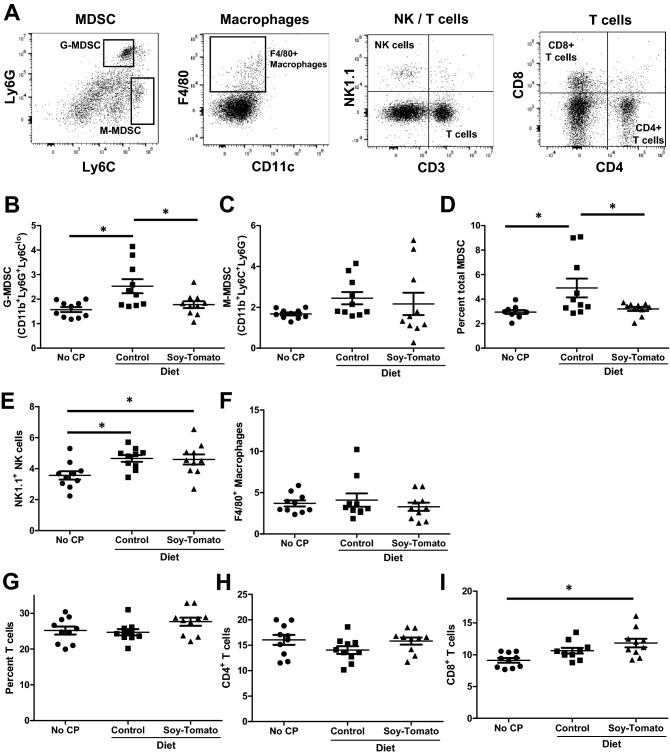
Soy-tomato diet modulates systemic immune populations in mice with CP. Splenocytes from healthy mice, mice with CP fed a control diet, or mice with CP fed a soy-tomato diet were stained by flow cytometry for circulating immune populations. (**A**) Representative plots illustrating gating strategy. Immune populations were quantified across the different cohort of mice identifying (**B**) CD11b^+^Ly6G^+^Ly6C^lo^ granulocytic (g)-MDSC, (**C**) CD11b^+^Ly6C^+^Ly6G^-^ monocytic (m)-MDSC, (**D**) total MDSC, (**E**) NK1.1^+^ NK cells, (**F**) F4/80^+^ macrophages, (**G**) total CD3^+^ T cells, (**H**) CD4^+^ T cells, and (**I**) CD8^+^ T cells.

Mice with CP have elevated levels of inflammatory and suppressive immune populations such as macrophages and myeloid-derived suppressor cells (MDSC). We analyzed spleens from each cohort of mice by flow cytometry to identify circulating immune populations (Fig. 7A). We observed significant increases in the percentage of splenic granulocytic-MDSC (*p* = 0.003), total MDSC (*p* = 0.008), and Natural Killer (NK) cells (*p* = 0.024) in mice with CP compared to mice without CP (Fig. 7B–E). Mice with CP fed the soy-tomato enriched diet showed significant reduction in the percentage of granulocytic-MDSC (*p* = 0.029) and total MDSC (*p* = 0.036) with no difference in monocytic-MDSC populations (Fig. 7B–D). No changes in macrophage or NK cell populations were observed when mice were fed a soy-tomato enriched diet compared to control fed mice (Fig. 7E–F). Lastly, we observed a trend in higher percentage of circulating T cells, specifically CD8^+^ T cells in mice fed a soy-tomato enriched diet compared to mice on a control diet or those without CP (Fig. 7G–I).

## Discussion

Compounds found in soy and tomatoes have shown to individually have anti-inflammatory effects in a range of diseases. This present study focuses on how a novel soy-tomato diet can modulate inflammation and reduce the severity of CP in a pre-clinical model. Utilizing the traditional murine model of chronic pancreatitis through continuous dosing of caerulein, we observed that a soy-tomato enriched diet reduces the severity of CP in mice compared to a control fed diet. We observed a reduction in systemic inflammation when mice were fed a soy-tomato enriched diet via reduced serum cytokine levels, reduced circulating inflammatory cell populations, and decreased number of T cells in pancreata. Importantly, these pathological and systemic inflammatory improvements were associated with improvements in physical activity patterns that were more favorable than CP mice on a control diet and comparable to mice without CP.

Our group has developed a novel soy-tomato juice that is currently undergoing clinical trials to determine whether it can reduce inflammation in patients with obesity. Thus, we were interested to test whether this soy-tomato diet could be effective in pre-clinical models of other inflammation-related conditions, such as CP. Recently we reported that in a small feasibility study that a soy bread intervention can reduce inflammation in patients with CP^32^. In the current study we observed similar decreases in cytokines such as IL-1β and TNF-α in mice fed a soy-tomato enriched diet. Interestingly, we also observed a significant decrease in serum IL-5 levels in mice fed a soy-tomato diet indicating this intervention may uniquely alter eosinophils driving CP inflammation. Eosinophilia can occur in patients with chronic pancreatitis and have an active role in tissue remodeling by activating fibroblasts, which increase collagen and fibrosis^33,34^. Eosinophils can be a potential source of inflammation-driven IL-5 production in patients with CP^35,36^. Tomato metabolites such as lycopene and β-carotene have previously been observed to reduce IL-5 and to suppress eosinophil function in other inflammatory conditions^37,38^. Additionally, we provide evidence that a soy-tomato enriched diet can reduce circulating MDSC and granulocytic-MDSC compared to control fed mice. MDSC can secrete cytokines such as IL-1β and IL-5 indicating a possible mechanism for the observed reduction in circulating levels in CP mice fed a soy-tomato diet^39,40^. We also observed changes in intra-pancreatic and circulating T cells in mice on a soy-tomato diet. Evidence suggests differences in central and effector memory populations in patients with CP compared to healthy individuals^41^. Future studies on how this dietary intervention modulates specific T cell populations in patients and whether MDSC have a role in this process will be of great interest. Additionally, MDSC in chronic pancreatitis could be a source for reactive oxygen species (ROS) which have been shown to be elevated and a cause of acinar destruction in chronic pancreatitis^42,43^. The ability of this soy-tomato dietary intervention to reduce inflammation in the pancreas could be due to a reduction in MDSC numbers thus decreasing local ROS in the pancreas microenvironment. This mechanism will be of great interest in future studies and trials with this dietary intervention. Interestingly, we observed a significant increase in circulating NK cells in mice with CP with no difference in numbers between control and soy-tomato fed mice. Increased NK cells in CP mice may exacerbate tissue damage and add to the inflammation. Our previous work provides evidence that soy isoflavones and their metabolites can reduce NK cell function^44^. Future investigation of a soy-tomato dietary intervention in patients with CP will study whether soy and tomato modulates NK cell numbers and their function.

Clinically there is no therapy available to reverse inflammatory damage associated with CP, so treatment is primarily focused on managing associated symptoms and complications. In clinical practice, pain treatment is guided by evidence from somatic pain studies with pain relief obtained from methods such as analgesic drugs, abstinence from alcohol and smoking, or endoscopy or surgery if there is evidence of an obstruction^45^. Therefore, novel therapeutics or dietary interventions that lessen inflammation in this disease could prevent many complications associated with CP and significantly improve quality of life for these patients, with a more favorable safety profile. In this report, we noticed mice with CP fed the soy-tomato diet appeared to be healthier, more actively moving in their cages, which suggests a potential reduction in pain than CP mice on a control diet. Rodents in pain generally present with a reduction in their activity and mobility^46,47^. To quantitatively assess whether the treatment diet reduced CP and thus pain in these mice, we monitored spontaneous activity over a 6 h period near the end of the study using an infrared-based monitoring platform. Mice with CP on a soy-tomato diet showed improvements in every activity measure tested compared to control diet-fed CP mice, similar to the activity levels of the baseline mice without CP. To our knowledge, this is one of the first reports to quantitatively measure activity to assess the health of mice with caerulein induced chronic pancreatitis. Currently there are no approved standard analgesics or anti-inflammatory drugs for patients with CP and few published data of their use to modulate pain and inflammation in rodent models of CP. Future studies should consider directly testing nociception assays in rodent models of CP to compare a soy-tomato dietary intervention to pharmacological interventions (such as anti-inflammatory drugs or analgesics).

Many questions still remain when considering the biological metabolites of soy and tomatoes in this novel diet and how they modulate inflammation and reduce CP severity. Major metabolites in soy like genistein, daidzein, and equol can have various effects on immunity whereas how metabolites from tomatoes like lycopene and naringenin affect inflammation is still poorly understood^44^. Breakdown and identification of the metabolites that are mediating the effect on CP will be important in future studies^21^. Carotenoids (lycopene) and flavonoids (naringenin, quercetin) are proposed to be the bioactive agents responsible for the health benefits of tomatoes and are posited to directly modulate inflammation^22–26^. Literature provides evidence that tomato metabolites such as lycopene, β-carotene, naringenin, and quercetin can inhibit inflammatory mediators COX and PGE_2_^48–50^. PGE_2_ and COX2 can mediate the differentiation of MDSC and promote inflammation^51^. In this study, we observed a significant reduction in circulating MDSC of mice fed a soy-tomato diet indicating that this dietary intervention may possibly target the PGE_2_/COX2 cellular pathway to reduce MDSC differentiation. How this soy-tomato dietary intervention and the associated metabolites effect MDSC differentiation will be of great interest in future investigations. Our group has developed a novel soy-tomato juice to use in patient trials which has previously been evaluated in healthy individuals for compliance, bioavailability, and effect on blood lipids^27^. Additional preclinical studies may be helpful to discover the specific bioactive compounds that mediated the effects seen in the current study. Furthermore, combined with favorable safety data and tolerability data from other studies, these results provide preliminary data supporting future studies on the anti-inflammatory role of a soy-tomato enriched diet in humans with CP.

## Methods

### Reagents and diet composition

Methanol, methyl *tert*-butyl ether, acetonitrile, water, hexane, acetone, formic acid and ammonium were of HPLC grade and purchased from Fisher Scientific. Beta-carotene was purchased from Sigma Aldrich, soy isoflavones (genistein, genistin, daidzein, daidzin, glycitein, and glycitin) were from ChromaDex, and lycopene was isolated and purified from tomato^52^.

### Diet formulation and analysis

Tomato powder was freeze dried from hot break tomato juice, made from high lycopene processing hybrids OH2461 and FG99-218 (both containing the old gold crimson (*og*^*c*^) allele, developed by David Francis at Ohio State University), and processed as previously described^53^. Tomato powder was incorporated into an AIN-93G diet at 10% w/w as previously described^54^. 0.7246 g of a 40% soy isoflavone (w/w) soy bean extract (Solgen 40 from Tradichem) was incorporated per 100 g total diet, substituting 7% corn oil, for soybean oil, in the AIN-93G diet formulation. The AIN-93G diet with 7% corn oil (in lieu of soybean oil) was used as a vehicle control (Control Diet). Carotenoids were extracted from mouse diets, analyzed using an Agilent 1260 ultra-high performance liquid chromatography with photodiode array detection (UHPLC-PDA) and lycopene and beta-carotene were quantified using authentic external standards^52,55^. Isoflavones were extracted from mouse diets, analyzed using HPLC–PDA, and daidzin, glycitin, genistin, daidzein, glycitein, and genistein were quantified using authentic external standards, modified from previously published methods^56^. Briefly, 0.5 g of diet was extracted with 4 mL of 60% acetonitrile, bath sonicated for 5 min, centrifuged for 10 min at 3000×*g* and supernatant removed. Addition of 60% acetonitrile, sonication, centrifugation and decanting was repeated 2 additional times, and supernatants were pooled. Aliquots were dried down, re-dissolved in methanol and analyzed using UHPLC-PDA on a Zorbax Eclipse (Agilent Technologies, 2.1 × 100 mm, 1.8 μm particle size) column, at 35 °C and with a gradient of A: water with 0.1% formic acid, and B: acetonitrile with 0.1% formic acid and flowing at 1 mL/min. The gradient was as follows with each ramp being linear: 10% B from 0–0.5 min, to 40% B from 0.5–2.5 min, to 45% B from 2.5–9 min, to 55% B from 9–11 min, to 100% B from 11–12 min, and a return to initial conditions for 2 min, for a total run time of 14 min. Analysis of tomato and soy compounds in control and soy-tomato diets are described in Supplementary Table 2.

### CP induction via IP caerulein injections

8 week old male C57BL/6 mice (Jackson Laboratories) were randomized and divided into control and experimental groups. Chronic pancreatitis was induced via intraperitoneal caerulein injections at 50 μg/kg. Caerulein is a cholinergic agonist, a cholecystokinin (CCK) analog derived from the Australian tree frog. CCK normally regulates exocrine pancreatic secretions after stimulation with food and repeated injections of CCK analogues like caerulein result in excessive acinar cell vacuolization, necrosis, and pancreatic injury in rodents^31^. Male mice were used as the severity of CP induced by caerulein is significantly greater than compared to female mice. The injections were administered hourly over the course of 7 h, twice a week for 4 weeks as previously described for mice to develop CP^30^. After 4 weeks of caeurlein injections, mice were fed a control or a soy-tomato enriched diet for 2 weeks. Baseline mice were continuously fed a control diet and injected with PBS as a vehicle control. An experimental group of no CP mice fed a soy-tomato diet was not included. We would expect no differences in mice without CP on a soy-tomato enriched diet as these mice are healthy and therefore would not have measureable changes in either inflammatory or disease outcomes selected for the current study. This is a similar design to the majority of dietary intervention studies, which do not include an experimental dietary group in healthy mice. However, when previously studied in other dietary interventions, there are no significant differences in food intake, toxicity, and health^54,57,58^. Following 2 weeks of dietary intervention, mice were weighed and then euthanized by CO_2_ asphyxiation and confirmed by exsanguination, and pancreata and serum were collected for analysis. Mouse body weights and food intake throughout the study are reported in Supplemental Fig. 1A,B. Serum levels of amylase and lipase were measured by spectrophotometric analysis using the Alfa Wassermann VetAce analyzer (West Caldwell NJ). Mouse drawing in Fig. 1A was created with Biorender.com.

### Quantification of histological acinar loss, fibrosis, T cell infiltration, and inflammation

Pancreata were formalin fixed and paraffin embedded prior to sectioning at 5 µm and staining with Hematoxylin and Eosin (H&E) or Masson’s Trichrome (MT). Pancreata were then evaluated for loss of acini, mononuclear inflammation, and stromal fibrosis using a modified chronic pancreatitis index (CPI) scoring system, as defined in Supplemental Fig. 3^59,60^. Loss of acini and mononuclear infiltrates were each determined in H&E stained sections by evaluation of ten 200 × magnification fields by light microscopy using an Olympus BX41 microscope (Olympus, Tokyo, Japan). Stromal fibrosis was assessed in MT-stained sections for each tissue section in total. The sum of scores was then determined to assign a CPI score.

### Immunohistochemistry

Immunohistochemistry for CD3 was performed by the Comparative Pathology and Mouse Phenotyping Shared Resource at the Ohio State University on a Thermo360 Autosatiner using a polyclonal rabbit antimouse CD3 antibody (#16669, IgG, Clone SP7, Abcam, Cambridge, UK) or isotype control. Briefly, the primary antibody was added at a 1:100 dilution to the slides and incubated for 30 min at room temperature, followed by incubation with the secondary antibody (1:1000, goat antirabbit, BA-1000, Vector Laboratories, Burlingame, CA) for 30 min and detection by using Vector ABC HRP RTU Elite (avidin–biotinylated enzyme complex, Vector Laboratories) with 3,3′-diaminobenzidine as the enzyme substrate for color development, according to the manufacturer's recommendations, then counterstained with Hematoxylin. Quantification of CD3 immunoreactivity was performed over five 400× magnification fields per tissue section and scored according to the total number of CD3 + cells within 5 fields (0 = absent, 1 = 1–5 cells, 2 = 6–49 cells, 3 = 50–100 cells, 4 =  > 100 cells).

### Activity monitoring analysis

Spontaneous physical activity was measured using an infrared-based sensing system (IR Actimeter and Actitrack software, Panlab, Harvard Apparatus, Holliston, MA). Four days prior to study endpoint, mice (n = 4) from each group were housed individually and cages placed inside 45 cm × 45 cm open-field arenas that emit a 16 × 16 grid of infrared beams. Breaks in the beams caused by mouse movements were translated into measures of activity. Mice were monitored for 6 h with ad libitum access to water, but diet was removed during activity monitoring as containers for the powdered experimental diets would interfere with the infrared beams. Data was compiled and analyzed for stereotype movement, locomotor movement, total activity, percentage of time resting, distance traveled (cm), and velocity (cm/s) for the duration of the 6 h.

### Cytokine and chemokine analysis from serum of mice

At study endpoint, serum was collected from blood via cardiac puncture. Cytokines and chemokines were analyzed from the serum using a Meso Scale Discovery biomarker multiplex assay (Meso Scale Diagnostics, Rockville, Maryland).

### Quantitative real-time PCR to determine expression of genes in the pancreas

At study endpoint, mice pancreata was harvested and placed in RNALater. Tissue samples were used to extract RNA using TRIZOL (Invitrogen) and reverse transcription reactions were performed using 400 ng RNA in a reaction with the high-capacity reverse transcription kit (Life Technologies). cDNA was used as a template to measure gene expression. TaqMan probes from ThermoFisher Scientific were used to assess *Il6* (Mm01210732_g1), *Il1b* (Mm00434228_m1), *Tnfa* (Mm00443258_m1), *tgfb* (Mm01227699_m1), *Ly6g* (Mm04934123_m1), and *Cd68* (Mm03047343_m1) gene expression which was quantified and normalized to the Eukaryotic 18S rRNA endogenous control housekeeping gene (Hs99999901_s1) and then normalized to healthy mice without CP.

### Analytical flow cytometry

Immunophenotypic analyses of splenocytes from mice were assessed by flow cytometry. Splenocytes were incubated on ice for 30 min with fluorochrome-conjugated antibodies, washed, and fixed in PBS containing 1% formalin for flow cytometric analysis on an Attune (Life Technologies) flow cytometer. Antibodies used to stain for murine surface markers: CD4 (Clone: GK1.5; Biolegend), CD8 (Clone: 53–6.7; BD Biosciences), CD4 (Clone: RM4-5; BD Biosciences), CD3 (Clone: 17A2; Biolegend), NK1.1 (Clone: PK136; BD Biosciences), CD11b (Clone: M1/70; Biolegend), Ly6G (Clone 1A8; BD Biosciences), Ly6C (Clone: Al-21; Biolegend), and F480 (Clone: N418; Biolegend).

### Statistics

Two-sample t-tests were used to compare chronic pancreatic index and CD3 infiltration between mice with CP fed a control diet and those fed a soy-tomato diet. A single mouse on the soy-tomato diet was determined to be an outlier in terms of CD3 infiltration and was removed from the analysis. Due to small sample size, exact Wilcoxon rank-sum tests compared expression levels of *Il6*, *Il1b*, *Tnfa*, *Tgfb*, *Ly6g*, and *Cd68*. Further, non-parametric Kruskal–Wallis tests with the corresponding Dwass, Steel, Critchlow-Fligner method to evaluate significance of pairwise comparisons was used to compare measurements of physical activity levels between the 3 mice groups. One-way ANOVA models with Tukey’s post-hoc tests were used to evaluate the remainder of the outcomes between the 3 groups where log transformations were taken as needed (IL-5, IL-6, IL-4, G-MDSC, Percent total MDSC, F4/80^+^ Macrophages).

### Ethics and animal approval

All animal protocols were approved by the Ohio State University Institutional Animal Care and Use Committee (IACUC) at The Ohio State University (Approved IACUC protocol 2009A0178-R3) and mice were treated in accordance with institutional guidelines for animal care. The Ohio State University Animal Care and Use Program is fully accredited by the Association for Assessment and Accreditation of Laboratory Animal Care International and follows Public Health Service policy and guidelines. All other experiments were completed under the research protocols (2014R00000086; 2013R00000056, 2017R0000073) approved by the Ohio State University Institutional Biosafety Committee.

## Supplementary information

Supplementary Information 1.

Supplementary Information 1.

## Data Availability

The datasets generated during or analyzed during the current study are available from the corresponding author on reasonable request.

## Abbreviations

AOX: Antioxidant
CP: Chronic pancreatitis
CPI: Chronic pancreatitis index
CXCL1: Chemokine (C-X-C motif) ligand 1
G-MDSC: Granulocytic myeloid derived suppressor cells
H&E: Haemotoxylin and eosin
IgG: Immunoglobulin G
IL: InterLeukin
IP: Intra-peritoneal
IR: Infra-red
Ly6G: Lymphocyte antigen 6
MDSC: Myeloid derived suppressor cells
MT: Masson's trichrome
NK cells: Natural killer cells
PBS: Phosphate buffer saline
TBHQ: Tert-butylhydroquinone
TGF beta: Transforming growth factor beta
TNF alpha: Tumor necrosis factor alpha
UHPLC-PDA: Ultra high-performance liquid chromatography-photodiode array
