# Supplementary material for: Soy-tomato enriched diet reduces inflammation and disease severity in a pre-clinical model of chronic pancreatitis
Source: Sci Rep. 2020 Dec 11;10:21824. doi: 10.1038/s41598-020-78762-9 (PMC7733503; doi:10.1038/s41598-020-78762-9)
Supplement: Supplementary file 1 — Supplementary Information 1. [file 41598_2020_78762_MOESM1_ESM.docx]

**Supplemental Figure 1. Confirmation of CP in mice injected with caerulein after 4 weeks of treatment.** 8 week old male C57BL/6 mice were injected intraperitoneally with 50μg/kg caerulein (7 hourly injections, twice weekly) for 4 weeks. A) Representative H&E and trichrome staining from PBS and caerulein injected mice after 4 weeks of treatment. B) Chronic pancreatitis index (score range 0-15) was assessed as previously described in Figure 1. Chronic pancreatitis was scored using these criteria: C) Loss of acini, D) mononuclear inflammation, E) stromal fibrosis, F) polymorphonuclear cells, and G) presence of necrosis.

**Supplemental Figure 2. Measurement of animal weight and food intake throughout *in vivo* study.** A) Body weight of mice from each group was measured throughout the experimental *in vivo* study. B) Control and soy-tomato enriched diet intake was measured during the final throughout the *in vivo* study. N=10 mice/group

**Supplemental Figure 3. Comparison of categories comprising the chronic pancreatitis index score.** A) Summary of the scoring method used to generate the chronic pancreatitis index score. Chronic pancreatitis was scored using these criteria: B) Loss of acini, C) mononuclear inflammation, D) stromal fibrosis, E) polymorphonuclear cells, and F) presence of necrosis.

**Supplemental Table 1. Composition of soy-tomato enriched diet for *in vivo* mouse studies.** 0.3% soy isoflavone extract and 10% tomato powder were added to an irradiated AIN-93G diet (Soy-tomato diet).  0.7246 g of a 40% soy isoflavone (w/w) soy bean extract (Solgen 40 from Tradichem) was incorporated per 100 grams total diet, substituting 7% corn oil, for soybean oil, in the AIN-93G diet formulation. The AIN-93G diet with 7% corn oil (in lieu of soybean oil) was used as a vehicle control (Control Diet).

**Supplemental Table 2. Analysis of tomato and soy compounds in control and soy-tomato diets.** HPLC was used to analyze the carotenoid and soy isoflavone compounds in the soy-tomato enriched diet compared to the control diet.

**Supplemental Table 1. Composition of Soy-tomato diet**

| **Grams/100 g Complete Diet** | | |
| --- | --- | --- |
| **Ingredients** | **Control Diet (AIN-93G)** | **Soy-tomato Diet** |
| Corn Starch | 39.7486 | 36.5034 |
| Casein | 20 | 18.2652 |
| Maltodextrin | 13.2 | 9.95 |
| Sucrose | 10 | 10 |
| Cellulose (fiber) | 5 | 3 |
| Corn Oil/Cottonseed oil | 7 | 6.5 |
| Mineral Mix | 3.5 | 3.5 |
| Vitamin Mix | 1 | 1 |
| L-Cystine | 0.3 | 0.3 |
| Choline Bitartrate | 0.25 | 0.25 |
| TBHQ, AOX | 0.0014 | 0.0014 |
| Tomato Powder | 0 | 10 |
| Soy Isoflavone Extract | 0 | 0.73 |
| **TOTAL (mg/100g diet)** | 100 | 100 |
|  |  |  |
| Total Isoflavones | 0 | 293.8 |
| Genistein/Genistin | 0 | 168.4 |
| Soy Protein | 0 | 72.3 |

|  | |  |
| --- | --- | --- |
|  | |  |
|  | |  |
|  |  |  |
|  | | |
|  |  |  |
|  |  |  |
|  |  |  |
|  |  |  |
|  |  |  |
|  |  |  |
|  |  |  |
|  |  |  |
|  |  |  |
|  |  |  |
|  |  |  |
|  |  |  |
| \| **Supplemental Table 2. Analysis of tomato and soy compounds in control and soy-tomato diets** \| \| \| \| \| \| --- \| --- \| --- \| --- \| --- \| \|  \|  \|  \|  \|  \| \| **Carotenoids** \|  \|  \|  \|  \| \|  \| **Compound** \| **mg/100g of Diet** \|  \|  \| \| Control Diet: \| Beta-carotene \| Not Detectable \|  \|  \| \|  \| Lycopene \| Not Detectable \|  \|  \| \|  \|  \|  \|  \|  \| \| Soy-tomato Diet \| Beta-carotene \| 0.13±0.01 \|  \|  \| \|  \| Lycopene \| 7.02±0.52 \|  \|  \| \|  \|  \|  \|  \|  \| \| **Soy Isoflavones** \|  \|  \|  \|  \| \|  \| **Compound** \| **mg/100g of Diet** \|  \|  \| \| Control Diet: \| *Total soy isoflavones* \| Not Detectable \|  \|  \| \| Soy-tomato Diet \| *Total soy isoflavones* \| 569.02±18.24 \|  \|  \| \|  \|  \|  \|  \|  \| \| Soy-tomato Diet \| **individual isoflavones* \|  \|  \|  \| \|  \| Daidzin \| 73.10±4.43 \|  \|  \| \|  \| Glycitin \| 108.39±2.90 \|  \|  \| \|  \| Genistin \| 378.51±15.86 \|  \|  \| \|  \| Daidzein \| 3.15±0.09 \|  \|  \| \|  \| Glycitein \| 1.86±0.29 \|  \|  \| \|  \| Genistein \| 4.02±0.17 \|  \|  \| \|  \|  \|  \|  \|  \| \|  \| **Total glycosides* \|  \|  \|  \| \|  \| Daidzin \| 73.10±4.43 \|  \|  \| \|  \| Glycitin \| 108.39±2.90 \|  \|  \| \|  \| Genistin \| 378.51±15.86 \|  \|  \| \|  \|  \|  \|  \|  \| \|  \| **Total aglycones* \|  \|  \|  \| \|  \| Daidzein \| 3.15±0.09 \|  \|  \| \|  \| Glycitein \| 1.86±0.29 \|  \|  \| \|  \| Genistein \| 4.02±0.17 \|  \|  \| |  |  |
|  |  |  |
|  |  |  |
|  |  |  |
|  |  |  |
|  |  |  |
|  |  |  |
