# Supplementary material for: Soy-tomato enriched diet reduces inflammation and disease severity in a pre-clinical model of chronic pancreatitis
Source: Sci Rep. 2020 Dec 11;10:21824. doi: 10.1038/s41598-020-78762-9 (PMC7733503; doi:10.1038/s41598-020-78762-9)
Supplement: Supplementary file 2 — Supplementary Information 1. [file 41598_2020_78762_MOESM2_ESM.pdf]

Supplementary Information

Soy-tomato enriched diet reduces inflammation and disease severity in a pre-clinical model of chronic pancreatitis.

Debasmita Mukherjee[1,*], Mallory J. DiVincenzo[1,2*], Molly Torok[1], Fouad Choueiry[1], Rahul Kumar[1], Anna Deems[1], Jenna L. Miller[3], Alice Hinton[4], Connor Geraghty[1], Jose Angel Maranon[5], Samuel K. Kulp[6], Christopher Coss[6], William E. Carson III[7], Darwin L. Conwell[8], Phil A. Hart[8], Jessica L. Cooperstone[3,9], and Thomas A. Mace[1,8].

[1]James Comprehensive Cancer Center, [2]Department of Veterinary Biosciences, [3]Department of Food Science and Technology, [4]Division of Biostatistics, College of Public Health, [5]Tradichem SL (Innovation center), Spain, [6]College of Pharmacy, [7]Department of Surgery, [8]Division of Gastroenterology, Hepatology, and Nutrition, The Ohio State University Wexner Medical Center, [9]Departments of Horticulture and Crop Science, The Ohio State University, Columbus, OH 43210

*Authors contributed equally to this manuscript

To whom correspondence should be addressed:
Thomas A. Mace, Ph.D.
Division of Gastroenterology, Hepatology, and Nutrition
The Ohio State University
420 W 12th Ave. Columbus, OH 43210
Tel: 614-366-3247
E-mail: thomas.mace@osumc.edu

Supplemental Figure 1

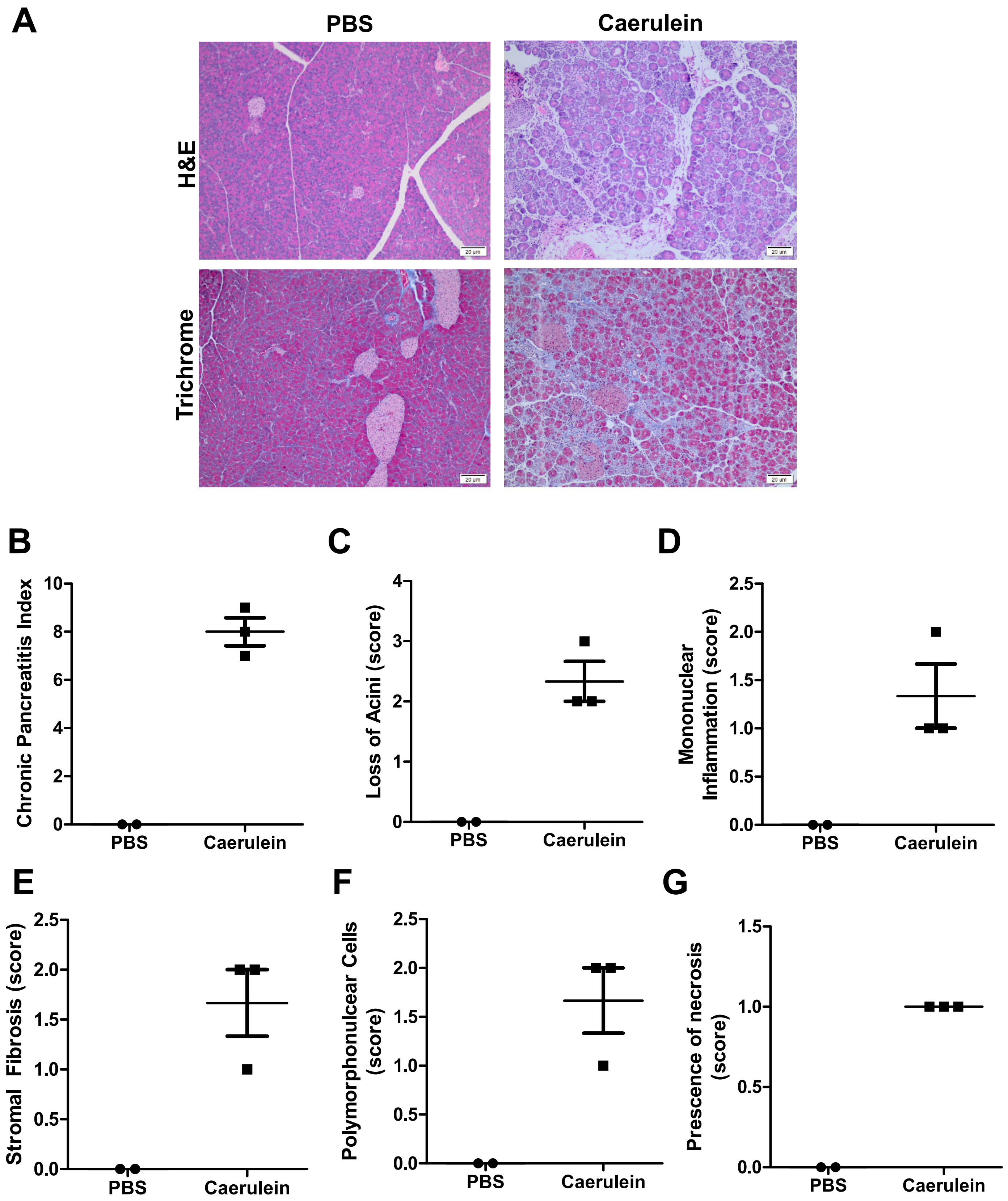

Supplemental Figure 2

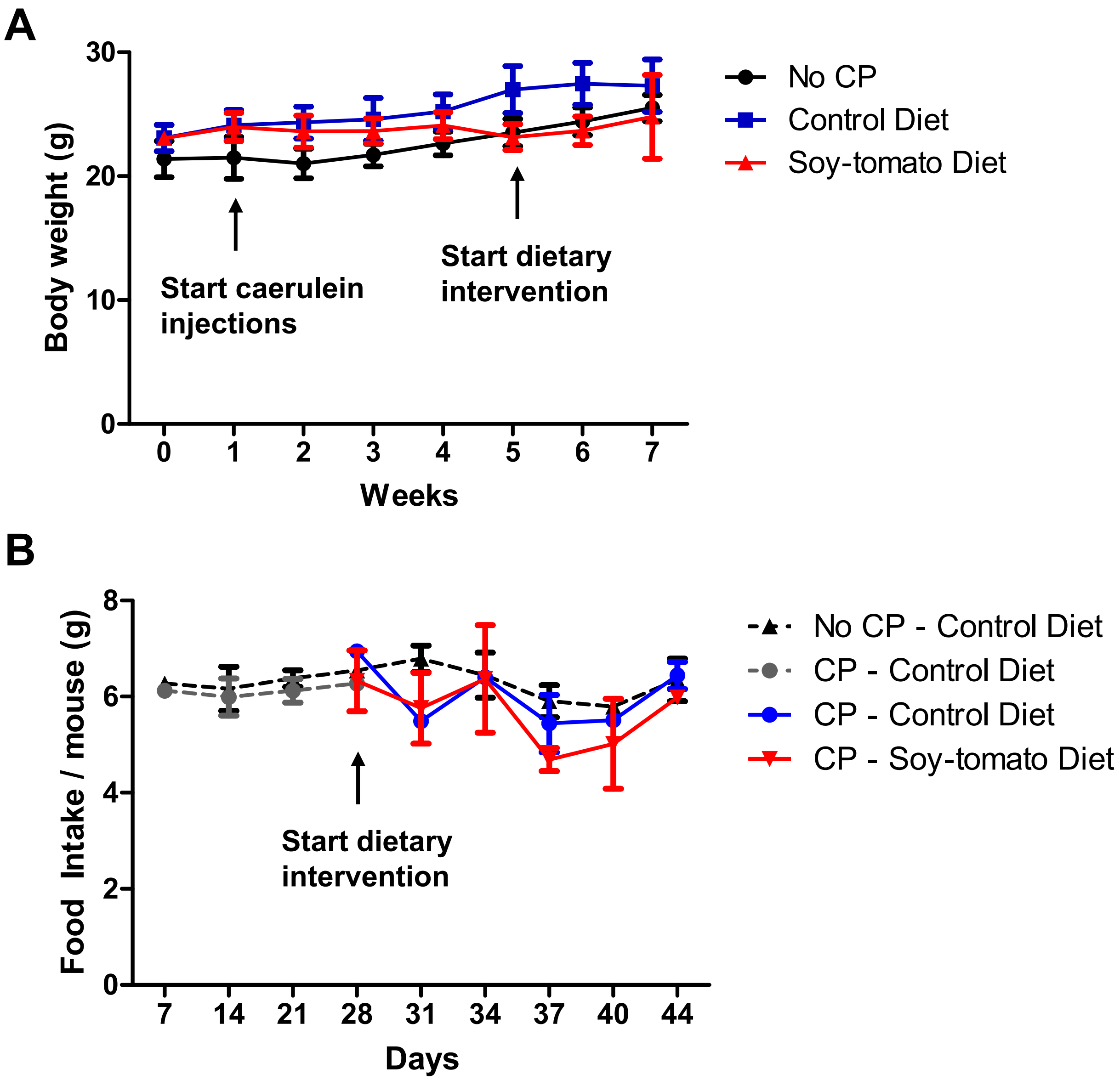

Supplemental Figure 3

A

Score	Loss of Acini	Mononuclear Inflammation	Stromal Fibrosis (Masson's Trichrome)	PMNs	Acinar Necrosis
0	Absent	< 5/HPF	Absent	Absent	Absent
1	< 10%	5-50/ HPF	< 5%	Minimal	Present
2	10-30%	50-100/HPF	5-15%	Mild	
3	> 30%	>100/HPF	>15%	Moderate	
4				Severe	

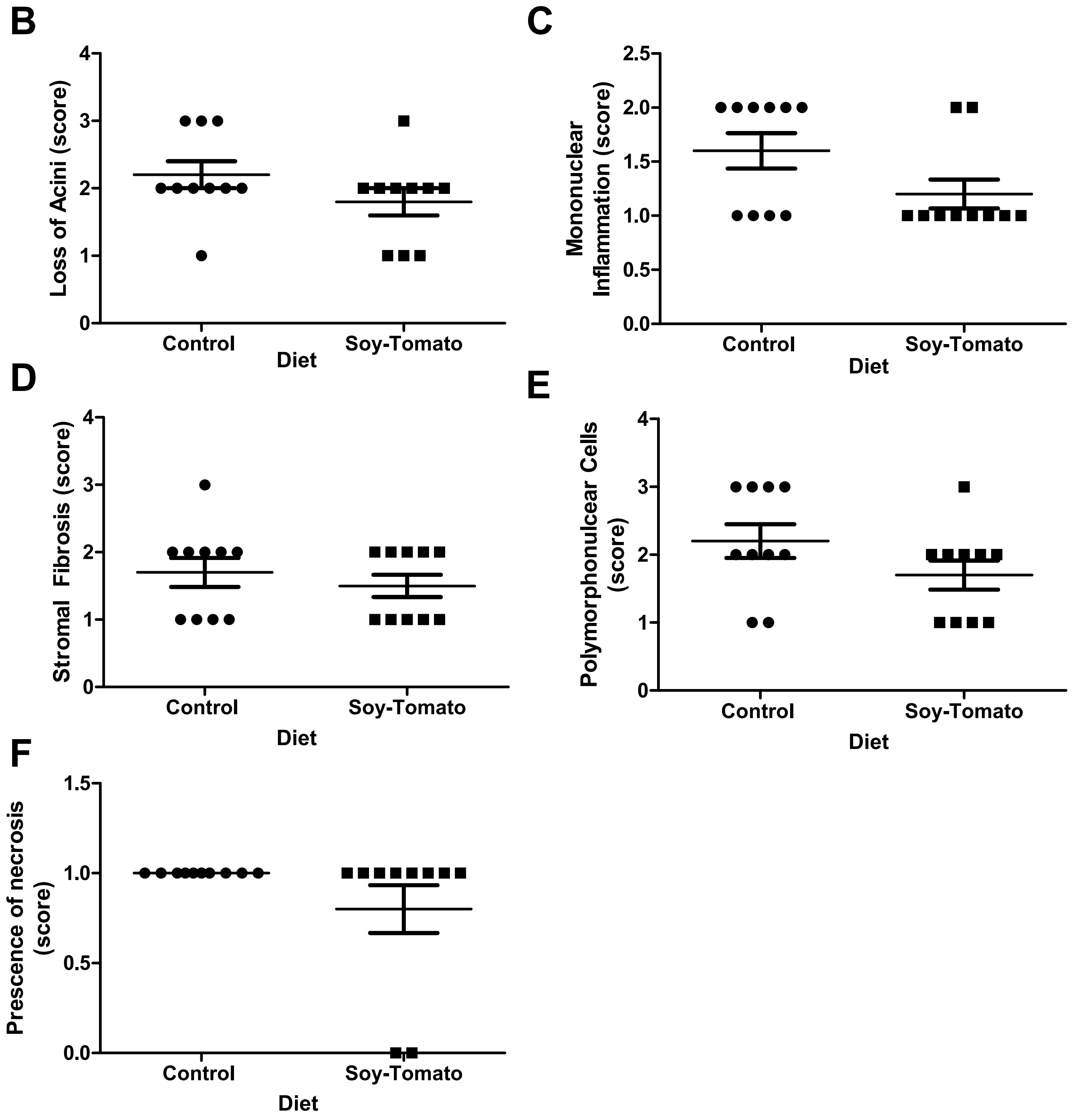

Supplemental Table 1. Composition of Soy-tomato diet

	Grams/100 g Complete Diet	
**Ingredients**	**Control Diet (AIN-93G)**	**Soy-tomato Diet**
Corn Starch	39.7486	36.5034
Casein	20	18.2652
Maltodextrin	13.2	9.95
Sucrose	10	10
Cellulose (fiber)	5	3
Corn Oil/Cottonseed oil	7	6.5
Mineral Mix	3.5	3.5
Vitamin Mix	1	1
L-Cystine	0.3	0.3
Choline Bitartrate	0.25	0.25
TBHQ, AOX	0.0014	0.0014
Tomato Powder	0	10
Soy Isoflavone Extract	0	0.73
**TOTAL (mg/100g diet)**	100	100
Total Isoflavones	0	293.8
Genistein/Genistin	0	168.4
Soy Protein	0	72.3

Supplemental Table 2. Analysis of tomato and soy compounds in control and soy-tomato diets

Carotenoids

	**Compound**	**mg/100g of Diet**
Control Diet:	Beta-carotene	Not Detectable
	Lycopene	Not Detectable
Soy-tomato Diet	Beta-carotene	0.13±0.01
	Lycopene	7.02±0.52

Soy Isoflavones

	**Compound**	**mg/100g of Diet**
Control Diet:	*Total soy isoflavones*	Not Detectable
Soy-tomato Diet	*Total soy isoflavones*	569.02±18.24
Soy-tomato Diet	**individual isoflavones*	
	Daidzin	73.10±4.43
	Glycitin	108.39±2.90
	Genistin	378.51±15.86
	Daidzein	3.15±0.09
	Glycitein	1.86±0.29
	Genistein	4.02±0.17
	**Total glycosides*	
	Daidzin	73.10±4.43
	Glycitin	108.39±2.90
	Genistin	378.51±15.86
	**Total aglycones*	
	Daidzein	3.15±0.09
	Glycitein	1.86±0.29
	Genistein	4.02±0.17
